# Tropical cyclones shape mangrove productivity gradients in the Indian subcontinent

**DOI:** 10.1038/s41598-021-96752-3

**Published:** 2021-08-30

**Authors:** Dina Nethisa Rasquinha, Deepak R. Mishra

**Affiliations:** Department of Geography, University of Georgia, Athens, GA 30602 USA

**Keywords:** Ecology, Climate sciences, Environmental sciences, Ocean sciences

## Abstract

Recent literature on the impact of cyclones on mangrove forest productivity indicates that nutrient fertilizations aided by tropical cyclones enhance the productivity of mangrove forests. We probe the implications of these predictions in the context of Indian mangroves to propose potential future directions for mangrove research in the subcontinent. First, we look at the time series trend (2000–2020) in satellite-derived gross primary productivity (GPP) datasets for seven mangrove forests across the country’s coastline. Second, we compare seasonal changes in soil nutrient levels for a specific site to further the arguments proposed in the literature and investigate the role of potential drivers of mangrove productivity. We find overall increasing trends for GPP over the past two decades for all seven mangrove sites with seasonal fluctuations closely connected to the tropical storm activities for three sites (Bhitarkanika, Pichavaram, and Charao). Additionally, organic carbon and nitrogen levels showed no significant trend, but phosphorus levels were higher during the post-monsoon-winter period for Bhitarkanika. Our findings expand the predictions of previous studies that emphasized the role of storm-induced nutrient fluxes and freshwater supply as primary drivers of productivity gradients in mangroves. Our study provides insights on how mangrove productivity may change with fluctuating frequency and magnitude of cyclones under a changing climate, implying the need for more mechanistic studies in understanding the long-term impact on mangrove productivity in the region.

## Introduction

The last few decades have witnessed a surge (~ 15% increase) in high-intensity tropical cyclones across the globe^1^. The increase in the proportion of high-intensity cyclones has significant implications for associated coastal systems through substantial socio-economic impacts. These increasingly frequent and intense cyclones can also have significant environmental consequences, including structural damages to ecologically sensitive coastal habitats such as mangrove forests, which provide many critical ecosystem services. In addition to their role in carbon sequestration and reducing water pollution, mangrove forests are well-known for their protective role against the dissipation of coastal storm surges. Mangroves and other coastal wetlands are considered as the first lines of defense against incoming cyclones. They help reduce the storm surge, wind shear, and the overall intensity of the cyclone and, in the process, also endure varying degrees of structural degradation such as defoliation and uprooting. The damages mostly result from strong winds, flooding, and the onslaught of runoff and sediment influx.

The Everglades, for example, have witnessed multiple significant hurricane events causing severe damage to the mangrove forests in the Everglades National Park. Hurricane Wilma damaged about 1250 ha of the Everglade mangroves in 2005, resulting in widespread stem mortality, basal area loss, and damage to the forest canopy, setting back the recovery that started following hurricane Andrew in 1992^2^. Similarly, in the Indian subcontinent, several recent tropical cyclones, namely Phailin (Category-5), Hudhud (Category-4), Fani (Category-5), Titli (Category-3), and Amphan (Category-4) are only a few notable examples of intense cyclones to make landfall on the east coast, since the great ‘Super Cyclonic Storm’ of 1999^3^. The latter devasted vegetation, human life, and property when it made landfall on October 19, 1999, in the state of Odisha on the eastern coast of the country. It killed around 9000–10,000 people, blew off structures, natural and man-made, with strong winds of 155 mph and a storm surge of about 8 m. Much recently, the country has witnessed several super cyclones on the east coast bringing back memories of 1999. According to an initial survey by the forest department of West Bengal, the super cyclone Amphan that hit the east Indian coast in May 2020 ravaged about 1600 sq. km of mangrove forests^4^.

The damages resulting from such annual cyclonic events are visually discernible through the physical destruction of habitats, trees, and property but the long-term ecological trends remain relatively less understood. For instance, a study by Castañeda-Moya et al.^5^ observed the positive aspects of hurricane impacts on the Florida Everglades mangrove ecosystem. They found that hurricanes help improve mangrove productivity, carbon assimilation, and resiliency due to vertical accretion from soil and nutrient deposition resulting from the land surface runoff. Mangrove ecosystems have high rates of carbon sequestration, which is reflected in their high aboveground biomass, soil carbon content, and belowground to aboveground biomass ratios^6–8^. Despite such high gradients of productivity, these systems are found to be nutrient deficient^9^. However, nutrient fertilization aided by tropical cyclones can enhance the productivity and growth of this typically nutrient-limited ecosystem. A single hurricane, Irma, in 2017 resulted in soil deposition 6.7–14.4 times greater than the past 100 years accretion rate^5^. Similarly, phosphorous (P) deposition contributed 49–98% to the total annual soil nutrient pool. The long-term interactions between mangrove forest productivity, tropical cyclone intensity, and resulting nutrient deposition, as well as its implications for coastal resiliency, remain uncertain. More so in developing nations like India, where mangrove ecology research is largely underrepresented.

**Figure 1:**
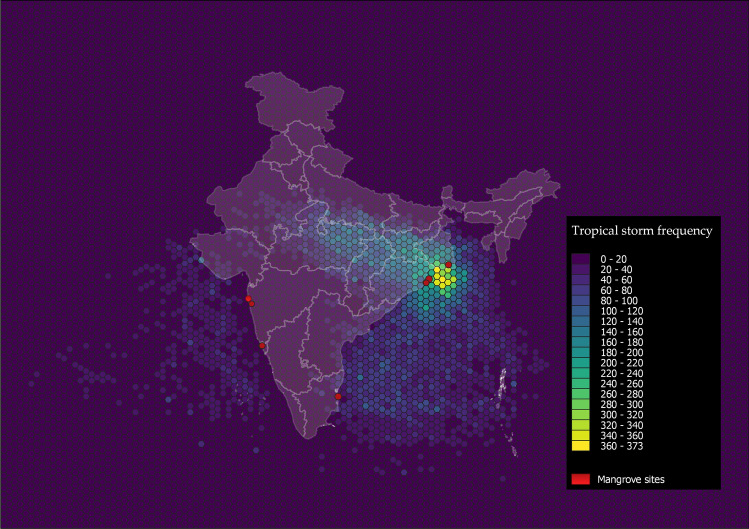
Cyclone frequency from 1890 to 2020 along the Indian coast. The numbers indicate the frequency of storms overlapping on a 0.5 by 0.5 deg hexagon grid. The darker purple shades represent low frequency, whereas green to yellow shades indicate higher frequency. Red circles show the location of mangrove forests explored in this study.

India possesses about 3% of the world’s mangrove forests which are shaped by press and pulse disturbances such as sea-level rise, coastal developments, and frequent tropical cyclones that impact the coasts annually (Fig. 1). These forests are distributed all along the west and east coast of the country, and along the low energy tidal fringes, estuaries, and lagoons of the Andaman and Nicobar Islands. The mangrove area extent, species diversity, and land use dynamics vary along the west and east coast. Along the east, the mighty Sundarbans in West Bengal (2112 km^2^) form the largest mangrove extent in the country followed by the island mangroves of the Andaman & Nicobar islands (616 km^2^)^10^. Other mangrove areas along the east coast include the river-fed mangroves of the Godavari and Krishna delta in Andhra Pradesh (404 km^2^), the high species diversity of Bhitarkanika and Mahanadi delta in Odisha (251 km^2^) and the estuaries of Pichavaram and Muthpet in Tamil Nadu (45 km^2^). In comparison, the mangrove area is much smaller and dispersed along the west coast, with the Gulf of Khambat and Kachchh covering the largest extent in Gujarat (1171 km^2^), followed by the urban mangroves of Mumbai and surrounding rural coastal belts in Maharashtra (320 km^2^), the bird haven of Charao in Goa (26 km^2^), the estuaries and backwaters in Kerala (9 km^2^) and the fringing mangroves of the Aghanashini and Kali rivers in Karnataka (10 km^2^)^10^.

Mangrove forest area, extent, floristic composition, and community diversity are vastly different between the east and west coast of India^11,12^. Similarly, the impact from cyclonic storms is also found to vary greatly, with the east coast experiencing more frequent and damaging storm events than the west coast (Fig. 1, Table 1). The differences in mangrove extent, species diversity, and productivity between the west and east coast can be attributed to the large estuarine environments shaped by the meandering deltas of the Ganga, Brahmaputra, Mahanadi, Krishna, Godavari, and Cauvery Rivers. The nutrient-rich alluvial soil and sedimentation from upstream discharge provide a conducive intertidal environment for mangrove colonization and growth. However, nutrient supply from upstream river flows can also be supplemented by nutrients through incoming stormwater pulses, which flood these regions more frequently than the west coast, annually (Fig. 1).

**Table 1:**
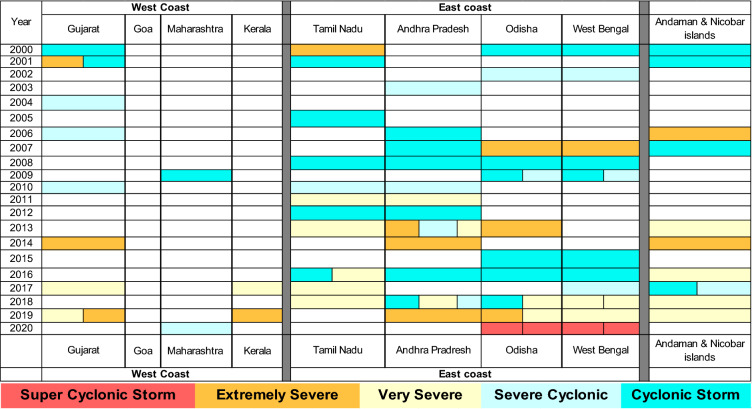
Time series of tropical storms across the east and west coast of India, including the Andaman Islands, compiled from data available through the Indian Metrological Department (IMD).

The colours represent the intensities of the cyclones in a given year. It does not, however, provide insight on the frequency of storms for any given year. Super Cyclonic Storm = Category 5; Extremely Severe = Category 4; Very Severe = Category 3; Severe Cyclonic = Category 2; and Cyclonic Storm = Category 1.

### Cyclone frequency and intensity along the indian coast

Between 1891 and 1970, a stream of about 98 tropical cyclones, including 55 severe ones, swirled the Arabian seawaters, whereas the Bay of Bengal absorbed and stood ground to nearly 346 cyclones, including 133 severe ones, in that same time period^13,14^. Studies have found that the losses occurring from cyclone events and even tsunamis were much less to areas protected by mangrove forest cover compared to other areas^15,16^. These relatively large numbers of cyclones swarming the Bay’s tidal and intertidal areas have only increased in the past few decades, as presented in Table 1.

The devasting super cyclone of 1999 that created havoc with several thousand deaths and destruction to property remains a distasteful memory following which studies investigated the role of mangrove vegetation in protecting coastlines against such fiery storm surges. One such study found that mangroves lining villages, especially those surrounded by large swaths of mangroves, experienced fewer deaths than those that were located directly in the tropical storm’s path^17^. Although the frequency and intensity of these extreme disturbance events that rile up the coast every year have been increasing over the past decades, the damages caused to human life and property have reduced drastically. That is because of the increased awareness, preparedness, and disaster management initiatives by local governments^18^. However, studies posit that the intensities of the disturbances shape the resilience of vegetation structure and function^8,19^. Studies also indicate an overall positive relationship between vegetation productivity and disturbance regimes, albeit at moderate levels^20–23^.

### Mangrove productivity gradients in the Indian subcontinent

Studies evaluating the biomass and carbon stock spatiotemporal dynamics of Indian mangroves remain few and far between, but that trend is slowly changing with greater value placed on the carbon storage potential of these forests. The Sundarbans, the largest mangrove area (approx. 2000 km^2^) in the country occupying more than 40% of India's total mangrove cover and a swooping floral diversity, both true and mangrove associate species, absorbs over 41.5 million tons of carbon dioxide daily, making it one of the most important blue carbon sinks in the world^15^. Similarly, mangrove forests in the Andaman Islands in the Bay of Bengal have shown to possess about 118.3 tC/ha of carbon stocks in biomass, followed by Tamil Nadu (62.81 tC/ha), Karnataka (50.40 tC/ha), and Gujarat (24.57 tC/ha) as documented by a series of studies compiled by Sahu et al. (2015). A much more recent study for Bhitarkanika and Mahanadi (Odisha) mangroves estimated a mean total of 124.11 ± 30.1 tC/ha^24^.

Consolidating these findings, we explored the gross primary productivity (GPP) trends across different mangrove sites in the country to understand the different drivers of change. Our central hypothesis for this study was inspired by Castañeda-Moya *et* al.^5^. We hypothesized that the cyclones and storm events have a net positive effect in the long-term on the mangroves' carbon assimilation abilities across the Indian subcontinent. We put forward two questions to test the hypothesis with publicly available data and model outputs.

1. Do these intense pulses of storm surges that bring in nutrients, sediments, and other debris, shape the long-term allocation of carbon in mangrove vegetation?
2. Are the differences in carbons stocks related to growth and productivity across India's west and east coast a function of mangrove extent and diversity, or do frequency and intensity of storm events influence mangrove vegetation growth and productivity gradients?

The goal of this study was to examine the long-term trends in GPP of Indian mangroves in reference to cyclone frequency and magnitude. We limited our investigation to seven different sites spanning five states across the west and east coast of India. These sites represent diverse protection regimes, species diversity, varying proximity to urban and agricultural centers, and a varying frequency of cyclonic storms.

## Results

### Long term GPP trends of Indian mangrove sites

**Figure 2:**
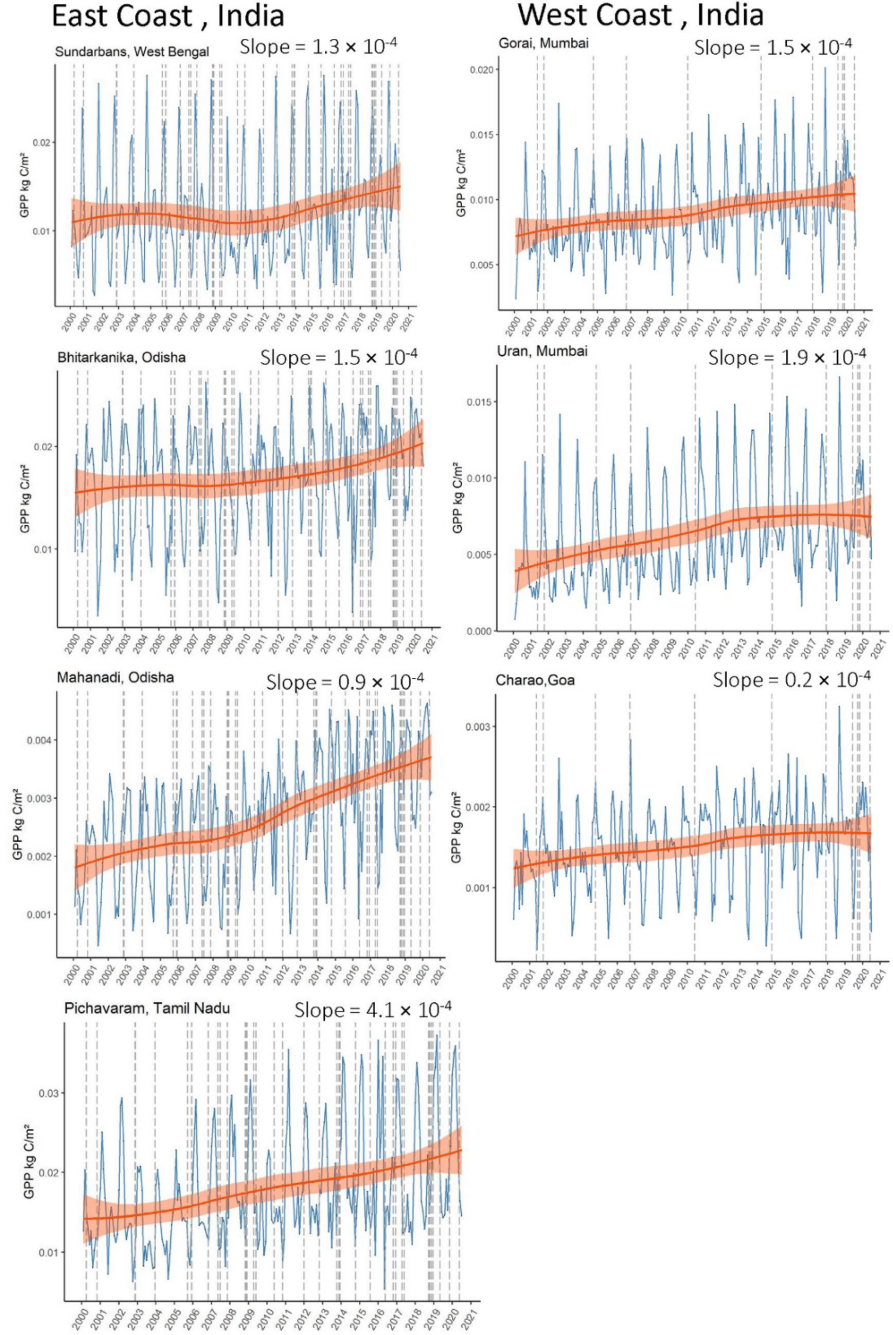
Time series of GPP for seven mangrove sites from Jan 2000 to July 2020. Dotted vertical lines indicate cyclone activity along the coast (number of tropical cyclones on the East coast, n = 39 and West coast, n = 12). Blue lines indicate the seasonal trend of GPP in a given year.

Our findings revealed the usual phenological cycle of GPP seasonally within any given year, but with a varying degree of an increasing trend over 20 years (Jan 2000 to July 2020) for all sites. Some sites, especially Pichavaram in Tamil Nadu, showed a remarkable increasing trend in GPP over the past two decades. Although no sharp trends were noticeable for the remaining sites, none showed a decrease despite the frequent and intense storms that have crossed paths with these forested areas, as shown in Fig. 2. Frequencies of cyclones on the east coast (n = 39) were relatively greater than those on the west coast (n = 12).

### Inter-site seasonal changes in GPP

Seasonal changes in photosynthetic rates of mangroves have also been noted in Bhitarkanka, Pichavaram, and Charao (Table 2). Of importance here is that post-monsoon and winter rates were found to be higher for east coast mangroves (Bhitarkanika and Pichavaram), whereas summer rates high for west coast mangroves (Charao).

**Table 2:** Seasonal photosynthetic rates, fPARChl, and LUE values for mangroves in Bhitarkanika (Area extent = 144.25 km^2^), Picharavaram (Areal extent = 7.86 km^2^), and Charo (Areal extent = 6 km^2^) for 2015^25^.

	Bhitarkanika (East Coast)			Pichavaram (East Coast)			Charao (West Coast)		
	Photosynthetic rate (g C m^−2^ day^−1^)	fPARChl	LUE (g C MJ^−1^)	Photosynthetic rate (g C m^−2^ day^−1^)	fPARChl	LUE	Photosynthetic rate (g C m^−2^ day^−1^)	fPARChl	LUE
Summer	1.2 (166,831)	0.049	3.93	2.6 (20,275)	0.15	2.20	7.7 (46,376)	0.064	5.24
Post-Monsoon	1.7 (248,762)	0.085	7.82	3.7 (29,255)	0.11	4.34	1.2 (10,658)	0.037	3.03
Winter	5.9 (844,486)	0.153	11.61	2.7 (21,560)	0.12	3.80	1.8 (7430)	0.049	1.77

LUE values are noted as mean values for dominant mangrove species on all sites. Values in parentheses represent the total overall GPP (carbon fixed per day) for each site.

**Figure 3:**
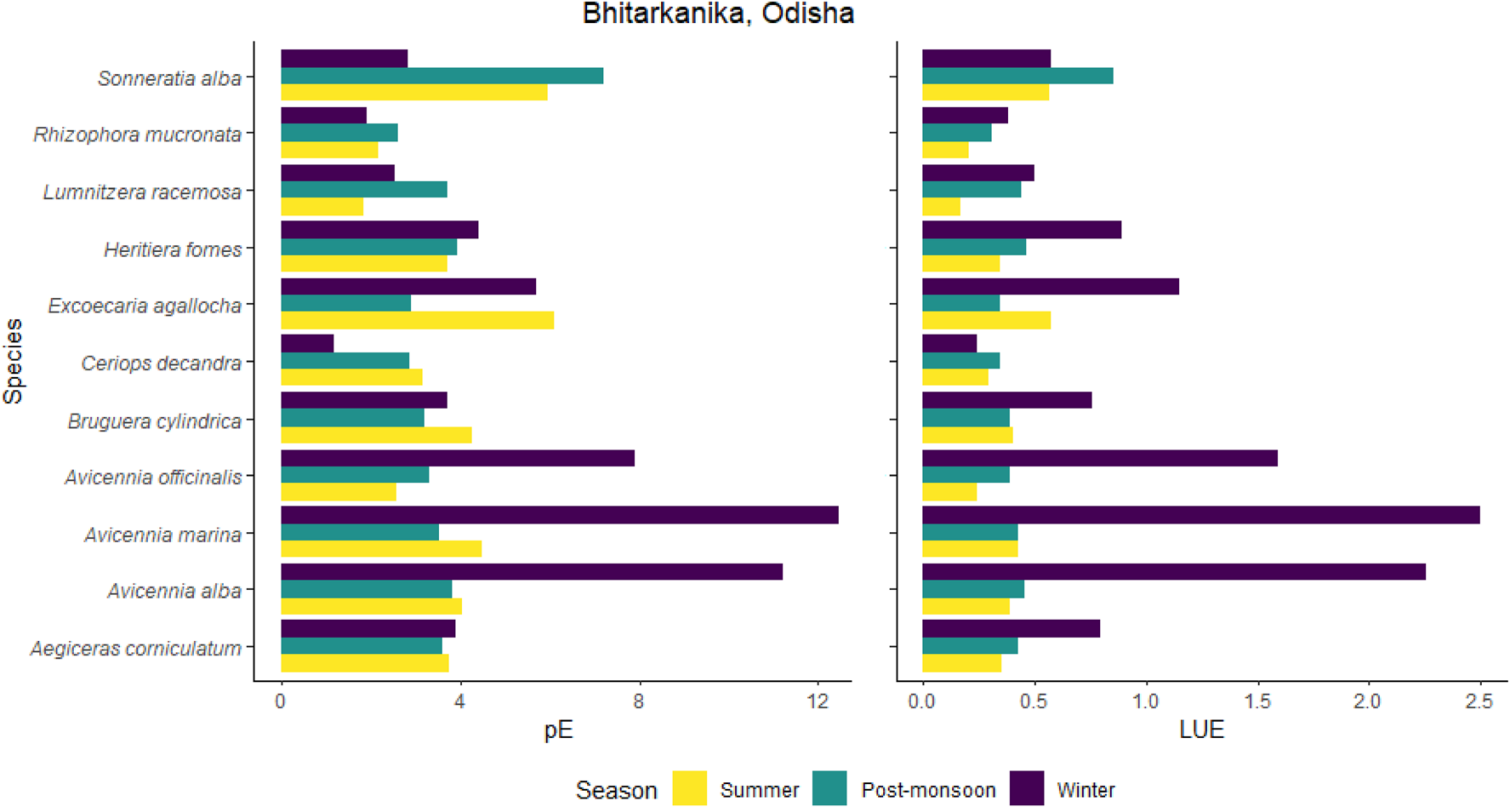
Seasonal variation in daily carbon fixation in terms of photosynthetic rate. (pE in g C m^−2^ day^−1^) and Light use efficiency (LUE or ɛ in g C MJ^−1^) in dominant mangrove species in Bhitarkanika adapted from a published Government of India report^25^. See SFig. 1 for Charao and Pichavaram.

Post-monsoon (7.82 g C MJ^−1^) and winter (11.61 g C MJ^−1^) LUE exhibited by dominant mangrove species (*Avicennia alba, Avicennia marina, Avicennia officinalis, Aegiceras corniculatum, Bruguiera cylindrical, Ceriops decandra, Excoecaria agallocha, Heritiera fomes, Lumnitzera racemosa, Rhizophora mucronata and Sonneratia apetala)* in Bhitarkanika were also found to be higher than in the summer (3.93 g C MJ^−1^). Among these species, the highest values were noted for *Avicennia marina,* which recorded a peak with 12.52 g C MJ^−1^ in winter, followed by *Sonneratia alba* (7.28 g C MJ^−1^) in post-monsoon and *Excoecaria agallocha* (6.17 g C MJ^−1^) in summer (Fig. 3). As LUE is directly proportional to GPP, we also see significant differences in GPP values between the summer and winter. For example, for the same recorded dominant species, GPP values for the summer (42.29 g C m^−2^ day^−1^) and post-monsoon season (41.00 g C m^−2^ day^−1)^ were significantly lower than what was observed for the winter season (58.03 g C m^−2^ day^−1^). Graphical outputs for Pichavaram and Charao are presented in the Supplementary Information (SFig. 1).

### Role of disturbance induced nutrient fluxes

**Figure 4:**
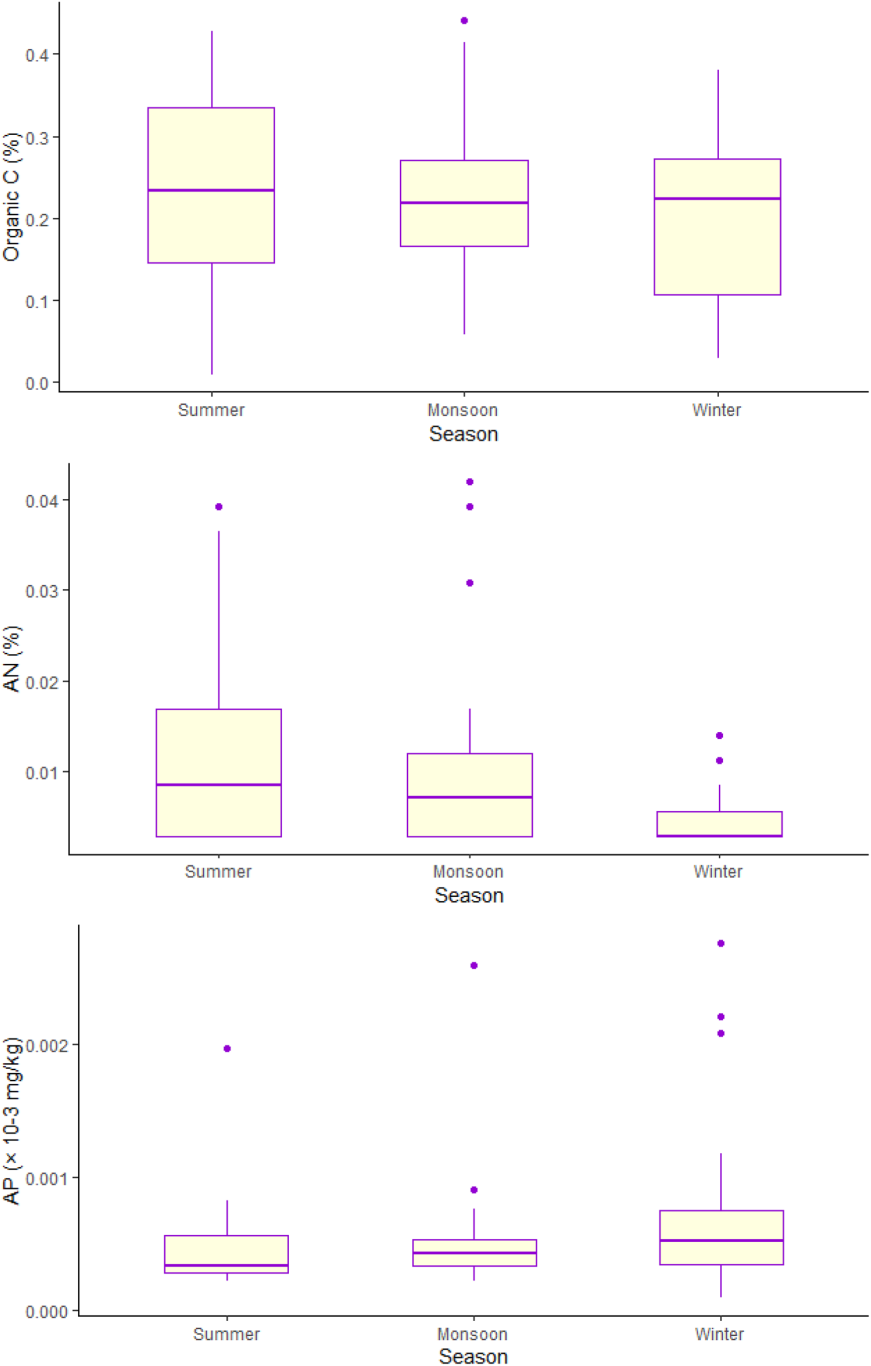
Soil carbon, available nitrogen, and phosphorus levels for the year 2015 for Bhitarkanika.

Apart from the physical damage caused by hurricanes and cyclones to mangrove stands, long term successional impacts through a regular supply of allochthonous sediments can also play a significant role in increasing surface elevation levels of these wetlands but at the same time also improve productivity and growth through timely nutrient pulses^2,5,9,26,27^. Although the data provided are limited, we find that seasonal changes in organic carbon and phosphorus levels, which is also a limiting nutrient for Indian mangrove wetlands^28,29^, tend to slightly increase in post-monsoon season (Fig. 4). However, nitrogen levels were lower in post-monsoon winter period as shown in Fig. 4, implying that more data would be needed to establish the seasonal dynamics of nutrient fluxes and their role in influencing GPP gradients.

Therefore, our initial question about, do GPP values increase because of nutrient fertilization caused by frequent tropical storms along the Indian coast, seems plausible but needs more investigation. Although the 20-year GPP time-series graphs do not show sharp patterns of increase for all sites, none of the sites showed a decline even though there was a clear indication of increasing frequency for storms in the past two decades. The seasonal differences are manifested in all sites, implying sudden spike of freshwater supply and/or nutrient availability post storm events in enhancing LUE and GPP trends (Bhitarkanika, Picharavarm, and Charao) which warrants the need for more such studies. Evidently, inter-site species differences also play a role in influencing GPP trends, as manifested in Fig. 3 and SFig. 1. Dominant species for each site can change the trajectory of recovery post-cyclone events, through differential competitive regeneration and growth processes.

### Drivers of productivity of Indian mangroves

**Figure 5:**
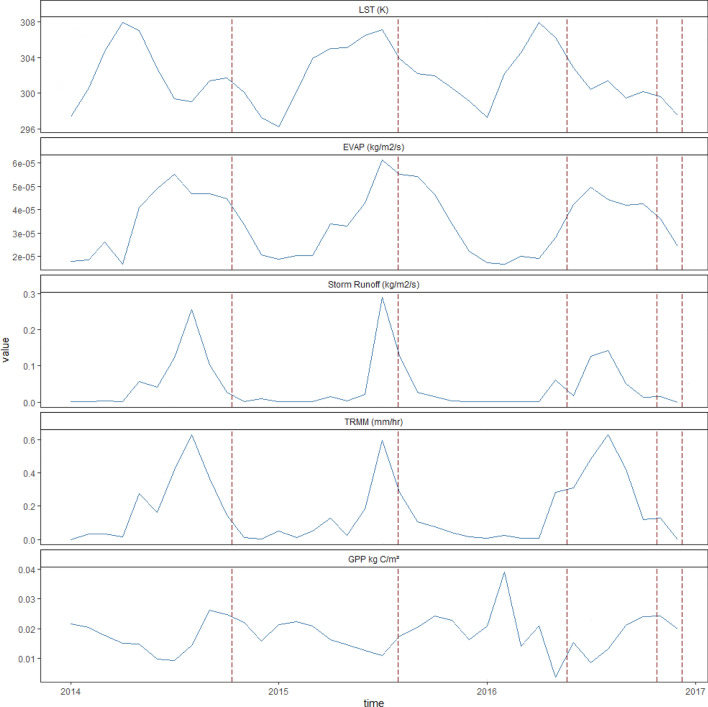
Time series, area-averaged monthly values for Land surface temperature (LST), Evapotranspiration (EVAP), Storm surface runoff, Precipitation Rate (TRMM), and Enhanced vegetation index (EVI) for pixels between Region 86.9014E, 20.6029 N, 87.0511E, 20.8007 N. LST data were obtained from MODIS-Terra MOD11C3 at 0.05 deg, EVAP data and Storm surface runoff values are model outputs of GLDAS-NOAH025 at 0.25 deg, precipitation rate data are from TRMM at 0.25 deg, and EVI values at 0.05 deg from MODIS-terra MOD13C2 satellite imagery.

Focusing again on the data-rich site of Bhitarkanika, and to better understand the drivers that influence mangrove GPP, we looked at possible relations between various meteorological variables and GPP. We find that GPP recovers slowly but steadily between 2015 and 2016 after previous storm events peaking in the first quarter of 2016 (Fig. 5). The spike in GPP is closely correlated with low evapotranspiration and LST values for the same period. Although, surface runoff and rainfall values dip during peak GPP, higher values are associated with storm events throughout. Also, worth noting is that during summer and pre-monsoon months^30^, ocean water inputs are found to be greater relative to river flow, leading to lower dissolved nutrient concentrations which can explain why GPP is influenced by storm surges. This also proves the trend in Fig. 5 for low evapotranspiration levels when GPP is high, implying low evapotranspiration levels maintain a steady flow of freshwater supply, keeping salinity in check, allowing GPP to recover post storms. However, recovery time is something that needs further investigation.

## Discussions

Productivity (GPP) of both terrestrial and coastal ecosystems is commonly estimated using the light use efficiency (LUE) model, which predicts that productivity is directly proportional to the amount of the incident photosynthetically active radiation (PAR). PAR is a function of seasons, latitude, and time of the day. This is particularly important as cloud cover in coastal areas can be variable, especially on the east coast, where it is found to be transient. In fact, coastal areas frequently experience overcast days, especially during cyclones and tropical depressions, which can influence light availability, and in turn, plant photosynthesis. Seasonal changes can also influence light availability and intensities, directly impacting photosynthetic rates of vegetation. Seasonal fluctuations in photosynthesis rates among other physiological parameters such as transpiration, PAR, and stomatal conductance were prominent in 16 true mangroves species in the Sundarbans^31^. For some mangrove species (*Aegiceras corniculatum, Avicennia officinalis, B. gymnorrhiza, and Xylocarpus mekongensis*), photosynthesis rates were considerably higher when measured in the winter than summertime despite lower levels of PAR (0.65 to 0.99 mmol m^-2^ s^−1^)^31^. According to several studies that found similar values^32,33^, it can be concluded that optimum PAR for mangroves is low, about 25–50% of full sunlight is sufficient to saturate photosynthetic rates in mangroves. Seasonality can thus play a crucial role in determining growth rates and productivity in mangroves by influencing photosynthetic rates, which are more prominent and species-specific during the winter season^31^.

Following this logic, the high post-monsoon and winter photosynthetic rates for the east coast mangroves can be attributed to the steady supply of freshwater from river discharge and monsoon rainfall which also varies between the northern and southern mangrove wetlands. For example, the southwesterly winds bring rains to Bhitarkanika in the monsoon season from June to September, as well as light showers during the northeast monsoon season running from October and December. This is also when Pichavaram experiences its monsoon season. Both regions experience a prolonged dry season which is longer for Pichavaram area extending from February to September. The number of extreme drought events have also increased considerably in this region over the years^34,35^. This has implications for the salinity gradients of the mangrove wetlands. For example, these gradients are found to be as high as 35 to 45 for Pichavaram. These prolonged dry spells are also found to be longer for southern mangroves than northern mangrove wetlands on the east coast, implying the role of tropical cyclones and depressions in influencing the flora, fauna, and fisheries of these mangrove wetlands.

Evidently, mangrove species diversity is recorded to be higher on the east coast (about 40 mangrove species belonging to 14 families and 22 genera) than on the west coast (27 species belonging to 11 families and 16 genera)^11,12^. Species-level differences can also be attributed to the differential ability of species to absorb and utilize available atmospheric CO_2_ as well as other macro and micro_-_essential nutrients necessary for growth. Some species (e.g., *Excoecaria agallocha* or *Sonneratia alba*) can use a high level of PAR effectively, whereas some species (e.g., *Avicennia marina or Avicennia alba*) have evolved in shady conditions and can thrive well only in a relatively small amount of PAR before photosynthetic rate levels off. Studies have shown that some species exhibit traits that increase resiliency to storm damage through large nutrient reserves, and plant tolerance to high salinity levels^8,23,36^. Some others, such as *Avicennia* sp. can easily re-sprout from epicormic shoots^8,36^ (Alongi 2008, Aung et al. 2013). A recent study conducted in China reports an overall positive impact of diversity on mangrove vegetation productivity and soil carbon storage levels^37^ (Bai et al. 2021). But disturbances through processes of gap-light dynamics can impact which species recover faster and contribute to the carbon pool. Disturbances can also enhance cryptic ecological degradation, compensating for the lost mangrove contributed biomass through mangrove associates that may have higher carbon storage levels^38^.

Frequent nutrient enrichment is the most plausible explanation behind the observed increasing trend of GPP in Indian mangroves, especially on the east coast. Though studies on Indian mangroves focus on dissolved nutrients, similar seasonal and annual trends in nutrient load post monsoon or tsunami induced events further our argument on nutrient induced fertilization changes in GPP^39–41^. Changes in organic phosphorus were found to increase by almost twofold following the tsunamic in 2004 in the Pichavaram mangrove ecosystem^39^. However, Alongi (2009 and 2014) reported that approximately 65% of GPP by the mangrove forest itself is respired, and the rest is partitioned into net primary productivity (NPP) allocated nearly equally to roots (36%), branches (32%), and foliage (32%). With an estimated carbon use efficiency (CUE) of only roughly 33%, a relatively small proportion of GPP is utilized in the aboveground biomass, and that could be why occasional physical damage such as defoliations due to cyclones and storms does not significantly impact the mangrove GPP. On the contrary, the negative impact of cyclones on canopy structure and canopy portion of the GPP, carbon assimilation by the mangrove forest as a whole is most likely being overcompensated by the increased pulse nutrient enrichment aftermath of the cyclones. That is because all other physical and meteorological variables, such as light, temperature, salinity, and tide level, that regulate GPP in mangrove ecosystems may have a seasonal (monsoonal) or intra-annual trend but do not reveal any interannual trend, unlike GPP in the past 20 years. Their relationships with mangrove LUE and GPP are complex and non-linear and do not have the compounding effect similar to nutrient deposition by increased cyclone frequency. In addition, Lovelock et al.^42^ have found that the proportion of GPP buried as C in the soil is higher for the fringing mangroves than fertilized mangroves because higher nutrient flow may speed up the decomposition of soil C hence decrease the C burial rates. That finding supports our observed 20-year GPP trend, which shows a net increase in GPP for all mangrove sites, and more interestingly, a slightly higher positive slope for east coast mangroves (See Pichavaram, Tamil Nadu), which are more frequently hit by cyclones compared to the west coast (Fig. 2). Based on these observations, we can theorize that the cyclones may be having a net positive effect on mangrove GPP by intermittent, excessive nutrient supply but could have an opposite effect by negatively affecting C burial rate and a declining soil organic carbon (SOC). We do not have data to examine the SOC trend for the same time period, but that could form a part of future research.

In summary, tropical cyclones interact with a range of biophysical drivers to influence productivity gradients of maritime forests, however, nutrient fertilization from frequent cyclonic storms and freshwater supply remain two critical primary indicators of observed GPP trends for all sites analysed in this study. The secondary drivers such as PAR, LUE, and other biophysical variables such as surface temperature, salinity gradients, rainfall, and evapotranspiration rates interact closely with each other to further influence GPP trends. In fact, mangrove sites in Asia are predominantly minerogenic, and sediment supply is essential for the long-term resilience of Asia’s mangroves^43^. This supply is strongly influenced by unpredictable rainfall regimes (or drought conditions), changes in hydrology (input of freshwater), flooding, and storm surge events which can further influence the growth and productivity of the forests by altering mangrove forest salinity gradients. For example, both higher salinity and greater seawater-sulfate concentrations have been found to decrease mangrove production^43^. Salinity levels also strongly influence competitive interactions among species^16^. For example, at Pichavaram, South India, mangroves are largely dying because of a combination of factors owing to hypersalinity, increasing temperature, low precipitation, and reduced tidal flushing^16^. Similarly, in Sundarbans, the reduction in freshwater inputs has resulted in a decrease in some species such as *Heritiera fomes* and *Nypa fruticans*^44^.

Although these drivers are inter-related and spatially variable through processes including sea level rise, changing ocean currents, precipitation, and temperature changes which influence evaporation rates, their synergistic impact can influence both mangrove growth and productivity levels. For example, studies show increases in precipitation values effect the increase in riverine discharge levels impacting a range of variables including sediment input levels, surface elevation and landward mangrove expansion rates ultimately leading to an overall increase in mangrove productivity^45–47^. In contrast, low precipitation and high temperatures increase evapotranspiration which can lead to increases in soil salinity and aridity, ultimately impacting seedling survival, species diversity, mangrove growth, and productivity over long time scales^46,48–50^. The complex interaction between environmental drivers is further influenced by tropical storms which is possibly the reason for the spike in GPP values post-cyclonic events. Tropical storms can temporarily decrease aridity-induced salinity changes, allowing nutrient fertilization to improve mangrove productivity levels. Equally noteworthy is how these drivers might interact with progressing climate change or increased nutrient load from land use change. The temporary spike in productivity levels might reach a break-off point and decline steadily or adapt to the changing climate to do better.

The exchange of CO_2_ between forests and the atmosphere has gained impetus in the last few years as forests can act as sink repositories to offset the steady growth of emissions. Although mangrove forests are increasingly seen as carbon-rich ecosystems because of their climate change mitigation potential^51–53^, knowledge and understanding of how mangroves in India may be influenced or changed because of climate change such as frequent intense cyclonic events is still insufficient. Over the past years, considerable efforts have been made in the field to understand the land-use change ecology of mangroves, ecosystem services evaluation, and natural history traits through a series of scientific studies. The studies have translated on-ground efforts into preservation, restoration, and afforestation efforts as coastal adaptation strategies. However, there remains much debate and contrasting views on the relationships between mangrove species diversity, composition, and productivity. Similarly, the trends in biophysical characteristics, dispersal ecology of these forests, and response to small-scale pulsed stressors such as grazing, insect herbivory, or extraction of forest products, also needs probing. More so the synergistic role of biophysical drivers on plant physiological traits and their future trajectory in the light of varied disturbances, need deeper examination for Indian mangroves.

## Conclusions and recommendations

Our synthesis of present knowledge on tropical cyclones impact on mangrove forests along the Indian coast highlights key conclusions which can direct future mangrove ecology research and provide a better understanding of potential future changes in mangrove productivity gradients with respect to change in frequency and intensity of cyclones. We conclude that (1) seasonal changes in GPP are influenced by tropical storms; (2) a change in nutrient levels can plausibly increase GPP levels post-tropical cyclones; (3) environmental drivers of mangrove productivity such as precipitation and evapotranspiration are heavily impacted by tropical cyclones on the Indian east coast influencing GPP gradients, whereas what drives productivity along the west coast needs further introspection and exploration.

In order to increase our knowledge of Indian mangrove ecosystem’s structure and function, we must go beyond empirical studies toward a mechanistic understanding of their life history. One way of addressing these gaps is to invest in the establishment of a network of Flux towers across the country to develop long-term measurements of CO_2_ fluxes at the soil–vegetation–atmosphere interface using the eddy covariance method (EC). EC measurements are useful in understanding how environmental controls such as climate, tidal activity and in turn salinity levels may interact with vegetation growth, productivity, and respiration at different times (hourly, daily, annually etc.), and scales^54^. On the other hand, rates of net primary production (NPP) may determine if mangrove forests are able to keep pace with sea-level rise. This can help understand the important feedbacks between forests and the atmosphere, ultimately shedding light on the response of vegetation to the changing climate.

These efforts could provide the improved understanding of mangrove ecology necessary to manage these forests at appropriate spatio-temporal scales and can be summarized as four key activities:

- Developing mangrove forest research and education field stations across the country.
- Increasing collaborations with national and local laboratory facilities and investing in laboratory capacity across field stations to conduct mangrove vegetation studies in the field.
- Training interested local communities in conducting field and lab research through accredited certification programs and appropriate compensation.
- Expanding open-source data sharing of mangrove research, deploying autonomous instruments and tools, and building research networks to collect and analyze mangrove ethnoecological data.
- Focusing scientific research surveys on mangrove vegetation in areas and at times where it is absent and where autonomous instruments cannot operate.

A combined effort across regional and local scales with local community stakeholders would provide the opportunity to integrate the generated data into novel models that consider both mangrove vegetation plot data in their biological and physical environment with global research efforts, enabling a holistic approach to ecosystem change research.

## Data and methods

We used a combination of satellite derived datasets to understand the trends in GPP across seven mangrove sites along the east and west coast of India. The satellite derived trends were then further analyzed to understand linkages with environmental and nutrient fluxes from recorded field observations from previous studies as described below.

### Cyclone frequency and intensity

Data for cyclone frequency and intensity was extracted from the International Best Track Archive for Climate Stewardship (IBTrACS), a global historical archive on tropical cyclones worldwide. IBTsACS provides data by collating cyclone track information from multiple regional sources^55^. We also referred to the Indian Meteorological Department (IMD) to understand the intensity of cyclones that have impacted the Indian coastline during the last 20 years.

### Long terms GPP trends across seven mangroves sites

We used MOD17A2H Version 6 500-m 8-day GPP products from MODIS (MOD17A2H, 500 m SIN Grid V006), which are developed using light use efficiency model and frequently used to monitor terrestrial and wetland carbon cycle processes^56^ to analyze time-series trends in mangrove forest GPP. The images were processed using the Google Earth Engine (GEE) platform and analyzed with R + RStudio. For each mangrove site, area-averaged GPP values were extracted from MOD17A2H 500-m pixels to understand trends with time. MODIS GPP products have been extensively used across the world in different forest types, including mangrove forests to monitor vegetation productivity patterns, including estimating changes in carbon sequestration over time, to understand how biophysical variables such as temperature or rainfall correlate with forest productivity, and to develop models to predict future productivity trends^57,58^.The high temporal frequency of the product makes it a reliable and feasible option while analyzing current and predicting future trends. Using MODIS GPP time-series plots (Fig. 2), we demonstrated the spatiotemporal trends in mangrove GPP for the seven Indian sites along the west and east coast, which are under a varying degree of cyclone disturbance regime.

### Seasonal GPP trends for three mangrove sites

While satellite derived GPP trends helped us understand long term GPP trends across the coastline, individual site analyses highlighted the seasonal dynamics of GPP with possible implications of the role of nutrients in influencing observed GPP trend. We collated data from published scientific reports on seasonal photosynthetic rates, fPARChl, and LUE values in Bhitarkanika, Odisha, Pichavaram, Tamil Nadu, and Charao, Goa for the year 2015 to investigate links between tropical storm activities and seasonal GPP trends^25^.

### Role of environmental variables and nutrients in influencing GPP trends

Further, to test if nutrient levels were higher post monsoon-winter seasons for the same time period (2015), and to understand the relationship between soil nutrient levels and changes in GPP, we analyzed soil carbon, nitrogen, and phosphorus data for Bhitarkanika forest mangrove site for the year 2015. Finally, we also looked at possible relations between various meteorological variables and GPP. Specifically, land-surface temperature (LST), storm runoff, evapotranspiration (EVAP), and rainfall patterns in influencing GPP gradients for a small mangrove region in Bhitarkanika (86.9014E, 20.6029 N, 87.0511E, 20.8007 N) using data from NASA's Giovanni (MEERA-2 simulations) portal for the years 2014–2016 as shown in Fig. 5.

## Supplementary Information

Supplementary Information.
