# Supplementary material for: Tropical cyclones shape mangrove productivity gradients in the Indian subcontinent
Source: Sci Rep. 2021 Aug 30;11:17355. doi: 10.1038/s41598-021-96752-3 (PMC8405614; doi:10.1038/s41598-021-96752-3)
Supplement: Supplementary file 1 — Supplementary Information. [file 41598_2021_96752_MOESM1_ESM.pdf]

Supplementary Information

Tropical cyclones shape mangrove productivity gradients in the Indian subcontinent.

Authors: Dina Nethisa Rasquinha[1*], Deepak R. Mishra[1]

Author affiliations:

[1] Department of Geography, University of Georgia, Athens, GA, 30602, USA

*Corresponding author email: drasquinha@uga.edu

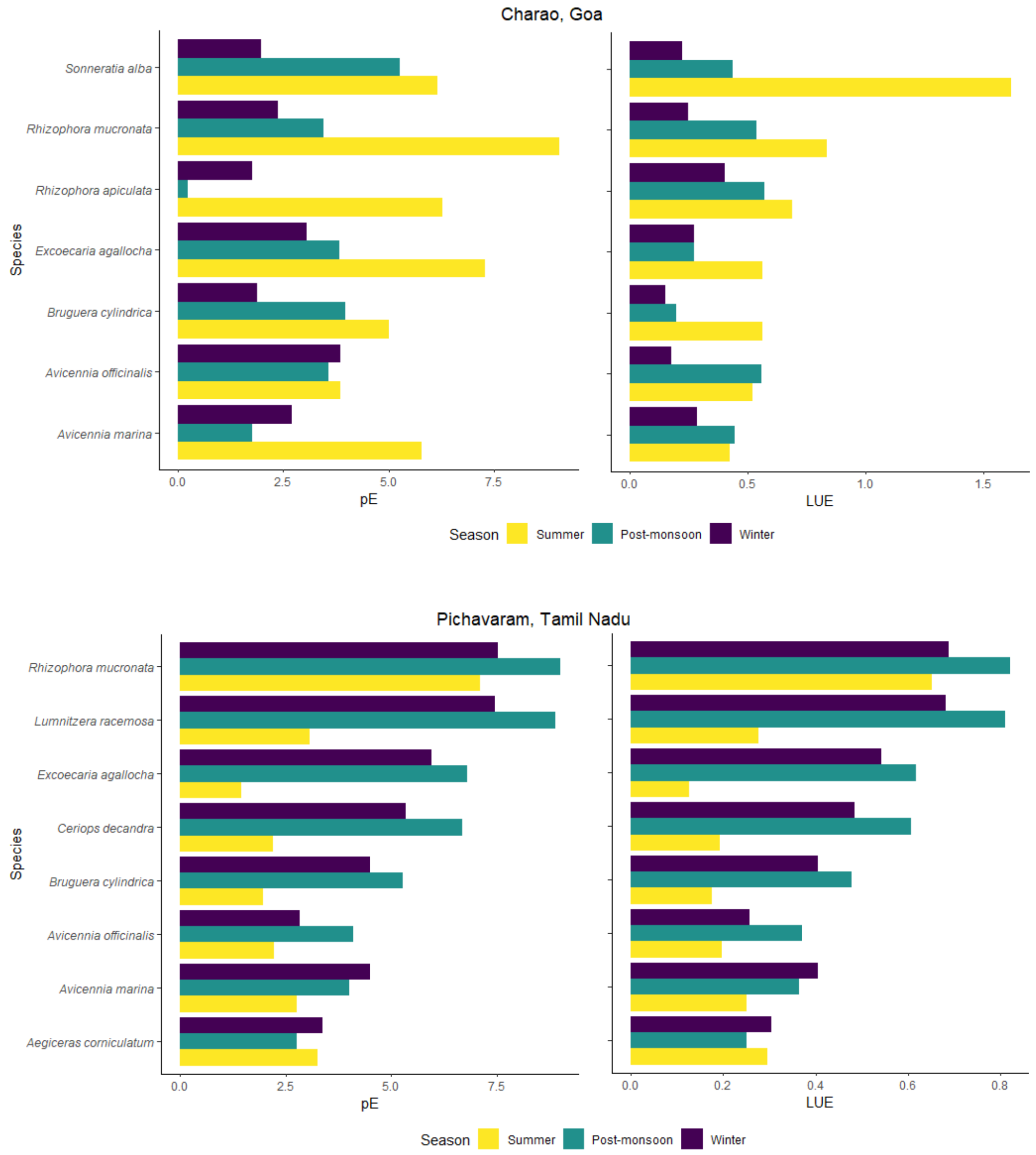

Figure S1: Seasonal variation in daily carbon fixation in terms of photosynthetic rate (pE in g C m^{-2} day^{-1}) and Light use efficiency (LUE or ε in g C MJ^{-1}) in dominant mangrove species in Charao and Pichavaram adapted from a published Government of India report[1].

References

1. TVR Murthy, N. V. L. and K. M. Biophysical characterisation and site suitability analysis for Indian mangroves. (2019).
